# Training the public health workforce for generative AI: validation of the Italian Chatbot Usability Questionnaire

**DOI:** 10.3389/fpubh.2026.1860473

**Published:** 2026-08-10

**Authors:** Francesco Baglivo, Aldo Gorga, Giuseppe Vella, Chiara Barbati, Antonella Lucia D'Atri, Matilde Pecchioli, Luigi De Angelis, Roberta Siliquini, Walter Mazzucco, Anna Odone, Caterina Rizzo

**Affiliations:** 1 Department of Translational Research and New Technologies in Medicine and Surgery, University of Pisa, Pisa, Italy; 2 Italian Society for Artificial Intelligence in Medicine (SIIAM - Società Italiana Intelligenza Artificiale in Medicina), Rome, Italy; 3 Department of Sciences of Public Health and Pediatrics, University of Turin, Turin, Italy; 4 Department of Health Promotion, Maternal and Infant Care, Internal Medicine and Medical Specialties (PROMISE) “G. D'Alessandro”, University of Palermo, Palermo, Italy; 5 Department of Public Health, Experimental and Forensic Medicine, University of Pavia, Pavia, Italy; 6 Medical Direction, Fondazione IRCCS Policlinico San Matteo, Pavia, Italy

**Keywords:** artificial intelligence, chatbot, digital health, healthcare professionals, prompt engineering, public health, training, vaccines

## Abstract

**Introduction:**

Artificial intelligence (AI) is becoming increasingly relevant for public health practice. However, scalable training models and validated usability instruments for AI chatbots are still limited in non-English contexts. We aimed to (i) assess AI knowledge, attitudes, and practices (KAP) among a sample of Italian public health professionals, and (ii) translate, culturally adapt, and psychometrically validate an Italian version of the Chatbot Usability Questionnaire (CUQ-IT) by applying it to a workshop-developed vaccination counseling chatbot.

**Methods:**

We conducted a three-phase study during the 57th Italian National Congress of Hygiene, Preventive Medicine and Public Health (Italy, October 2024): (i) use of an Italian translation and adaptation of the CUQ within a cross-sectional KAP survey; (ii) a 90-min theoretical-practical workshop with guided prompt engineering and collaborative development of a Custom GPT for vaccination counseling (VaxSense), followed by supervised interaction; (iii) post-workshop usability evaluation with CUQ-IT and psychometric testing. Construct adequacy was assessed using KMO and Bartlett's test. Internal consistency was estimated with Cronbach's α. Associations between KAP and usability ratings were explored.

**Results:**

Of 150 workshop attendees, 87 returned questionnaires (58%); 86 were eligible for KAP analyses, and 77 completed CUQ-IT for usability evaluation. Participants (mean age 35.9 ± 8.7; 55.8% females) reported moderate AI knowledge (mean 2.74 ± 1.05/5), positive attitudes (3.87 ± 0.73/5), and limited routine use (2.88 ± 1.11/5). CUQ-IT showed good sampling adequacy (KMO = 0.81) and significant item correlations (Bartlett's χ^2^ (120) = 515.36, *p*-value <0.001). Internal consistency was high overall, with Cronbach's α = 0.881. The mean CUQ-IT usability score for VaxSense was 66.60 ± 15.29 (range 18.75–95.31). Participants with higher AI knowledge, more positive attitudes, and more frequent AI use tended to assign higher usability scores.

**Conclusion:**

In our sample of Italian public health professionals, moderate baseline AI knowledge, positive attitudes, and limited routine use of AI emerged, while the workshop-developed vaccination chatbot achieved overall good usability. The significant gender differences observed in both self-rated AI knowledge and usability scores indicated that “AI readiness” may be uneven, even within trained professional groups, underscoring the need for inclusive capacity-building strategies. In the public health sector, training should be framed as a component of safe implementation: strengthening the ability to critically appraise outputs, recognize common failure modes, mitigate bias and equity risks, and apply privacy-by-design principles. CUQ-IT demonstrated robust psychometric performance and represents a standardized tool to benchmark chatbot usability in Italian settings. Future work should confirm its measurement structure in larger samples and assess whether targeted training may reduce capability gaps and support appropriate and sustained use of AI in public health practice.

## 1 Introduction

Public health practice and research are undergoing a digital transformation, with artificial intelligence (AI) poised to revolutionize surveillance, health communication, and decision support (1). As these technologies evolve, the public health workforce's capacity to harness their potential while mitigating risks related to bias, misuse, and inequitable implementation depends critically on upskilling (2, 3).

In recent years, international recommendations have converged on the need to strengthen AI literacy and foster practical skills among public health professionals (4, 5). There is a broad consensus on the need to integrate theoretical knowledge with practical experience, especially when dealing with generative AI tools (6). However, examples and guidance on how to realize such initiatives remain limited. Recent empirical evidence reinforces the urgency of strengthening AI training, as physicians worldwide continue to report limited knowledge, mixed attitudes, and insufficient practical experience with AI-enabled digital health tools (7). Inadequate training is reported as a major barrier to safe implementation. Importantly, those with prior exposure or hands-on experience with AI exhibit more positive attitudes, fewer concerns, and greater readiness to adopt AI in practice (7).

As generative AI-powered chatbots are increasingly proposed for use in public health practice, their systematic evaluation and validation become essential to ensure safety, effectiveness, and appropriateness for real-world implementation (8–10). Evidence supports the effectiveness of such tools in enhancing health literacy and promoting positive health behaviors, particularly in vaccination contexts where they may be able to counter misinformation and strengthen patient-provider communication (11, 12). However, chatbots may exhibit variability in usability, transparency, reliability, and potential for unintended harm, particularly when deployed in complex public health settings. Robust evaluation frameworks are therefore required to assess not only technical performance, but also user-centered dimensions such as clarity, trustworthiness, cognitive load, and perceived usefulness (13).

The Chatbot Usability Questionnaire (CUQ) represents a standardized instrument specifically designed to assess the usability of AI-powered conversational agents (14). Importantly, the meaningful application and interpretation of usability instruments such as the CUQ require a minimum level of AI literacy and practical experience, as users must critically engage with chatbot behaviors, limitations, and outputs to provide valid and informative evaluations (14).

From a public health perspective, equipping professionals with the skills needed to both use and critically assess AI-powered chatbots is a prerequisite for their responsible adoption. Without adequate training, usability evaluations risk being superficial or biased, potentially overlooking issues related to misinformation, automation bias, or inequitable user experiences. Conversely, hands-on exposure to generative AI tools may help practitioners to develop the experiential knowledge necessary to meaningfully contribute to validation efforts and to interpret usability metrics in light of real-world public health needs (15).

In this context, two key needs emerged: training public health professionals to enhance their AI literacy and validating an instrument to assess the usability of AI-powered chatbots. To address these needs, the Italian Society of Hygiene and Preventive Medicine (SItI), in collaboration with the Italian Society of Artificial Intelligence in Medicine (SIIAM), held a theoretical-practical workshop during the 57th National Congress of Hygiene, Preventive Medicine and Public Health, which took place in Palermo from 23 to 27 October 2024. The workshop titled “Digital Public Health: The Role of Large Language Models in the Future of Public Health, State of the Art and Practical Implementations” was designed to provide participants with both theoretical information regarding generative AI and hands-on experience in developing and testing generative AI tools.

This study reports the qualitative and quantitative outcomes of the workshop, analyzes participants' knowledge, attitudes, and practices regarding AI, and presents the psychometric validation of the Italian version of the CUQ.

## 2 Materials and methods

**Figure 1:**
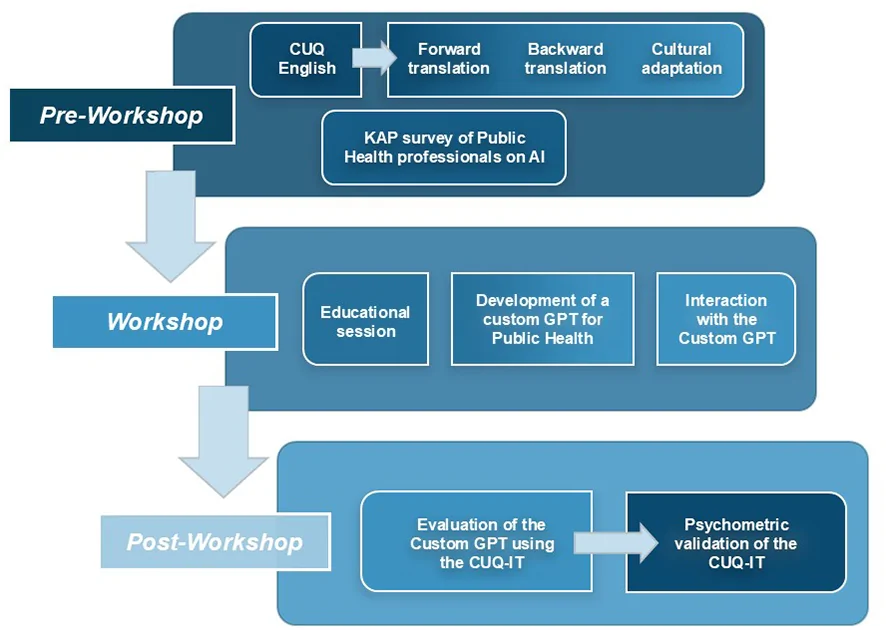
Flow diagram of the 3 phases of the study.

The study design can be divided into 3 phases, as described in Figure 1 and specified as follows:

(i) pre-workshop, including

(a) the translation and cultural adaptation of the CUQ in Italian, and
(b) a cross-sectional survey assessing knowledge, attitudes, and practices (KAP) of public health professionals toward AI;

(ii) a theoretical and practical workshop;
(iii) a post-workshop

(a) evaluation of a generative AI tool developed during phase ii, and
(b) the psychometric validation of the Italian version of the CUQ (CUQ-IT) based on participants' answers.

### 2.1 Participants and inclusion criteria

Participants were recruited through voluntary enrollment using a convenience sampling strategy. The invitation to participate was extended to all attendees of the aforementioned workshop on the 25th October 2024. To maintain anonymity and encourage candid responses, no identifying information was collected. Inclusion criteria required participants to (i) hold a degree in medicine or a health-related profession, and (ii) provide informed consent. Individuals were excluded if they declined to provide consent.

### 2.2 Translation and cultural adaptation of the CUQ in the Italian language

The Chatbot Usability Questionnaire (CUQ) is a validated instrument designed to assess chatbot usability, including characteristics such as personality, onboarding, understanding, error handling, and intelligence (16). It was developed in English by a team from Ulster University and lacked a validated Italian translation. The CUQ consists of 16 balanced items covering multiple dimensions of chatbot usability, including efficiency, ease of use, understanding, and user frustration, with eight positively framed and eight negatively framed statements. Responses are aggregated into a total score ranging from 0 to 100, enabling quantitative comparison across tools and contexts.

We produced the Italian version of the CUQ, called CUQ-IT, following internationally recognized procedures for the linguistic and cultural adaptation of psychometric instruments, including forward-backward translation and psychometric validation, to ensure clarity, cultural relevance, and reliability (17).

Regarding the translation process, two of the authors (AG and GV) independently produced the initial translation and cultural adaptation from the source language (English) into the target language (Italian). Subsequently, two additional authors (CB and LDA) back-translated the text into the original language. A third author (FB) reviewed the two versions and resolved any discrepancies, ensuring maximal fidelity to the original meaning and cultural relevance.

### 2.3 KAP cross-sectional survey on artificial intelligence for public health professionals

Before the workshop started (phase i), we conducted a cross-sectional survey. The survey was paper-based, self-administered, anonymous, and voluntary. It comprised five items: three single self-assessment items measuring knowledge, attitudes, and practices regarding AI in healthcare, and two further items (perceived usefulness of AI-specific training events and willingness to use an AI-based system to support professional decisions) addressing constructs outside the scope of this study. All five items were Likert-scale items ranging from 1 to 5, where 1 was the lowest and 5 the highest, for each measured domain.

Regarding the three items to evaluate knowledge, attitudes, and practices, we adopted the following definitions: by knowledge, we referred to the participants' understanding of what AI is, the principles underlying its functioning, and its applications and tools in the healthcare domain. By attitude, we meant the perception of such tools and their evaluation in the context of daily professional practice. By practice, we referred to the frequency with which AI-based tools are used in routine professional healthcare activities. These were *ad hoc* single-item measures developed for this study rather than a previously validated multi-item scale; as each construct was assessed by a single item, internal-consistency testing was not applicable. The knowledge item captured self-rated familiarity with AI rather than objectively assessed knowledge.

### 2.4 Theoretical and practical workshop

The workshop was designed and delivered by a team of public health physicians and researchers with expertise in the application of artificial intelligence to public health and medicine, affiliated with the Italian Society of Hygiene and Preventive Medicine (SItI) and the Italian Society of Artificial Intelligence in Medicine (SIIAM). It lasted 90 min and comprised three sessions to balance theoretical learning with practical application:

1. **Educational session**. This session comprised three talks of about 15 min each, the first one by CB titled “Basic understanding and potentialities of large language models in public health” to give participants basic principles of different types of artificial intelligence (machine learning, deep learning, generative AI, etc.), use cases in public health from the literature, and general guidance on biases and ethics. The second one by FB, titled “Practical Applications of LLMs and AI Chatbots in Public Health: Beyond ChatGPT,” focused on the use of generative AI in public health, discussing the concept of prompt engineering and focusing on the basic principles of interaction with generative AI models, including the garbage-in, garbage-out concept. The last talk by GV on “Developing Personalized AI tools for public health, the example of Custom GPTs,” focused on the process of creating a personalized chatbot using ChatGPT Custom GPTs builder, including key concepts of knowledge selection for retrieval augmented generation (RAG), system prompt design, and general instructions to align a generalized model (in this case a GPT) to a specific task (in this case a Public Health related task).
2. **Hands-on AI tool development session**. This session lasted approximately 30 min, was conducted by AG, and included an interactive component in which participants collaboratively designed a Custom GPT using OpenAI's GPT Builder. Through a structured process with multiple-choice decision points, they defined the chatbot's purpose, refined its communication style, specified areas of focus, and tested its capabilities.
3. **Interaction with custom GPT**. After collaboratively designing Custom GPT, it was publicly shared via QR code with participants, who were asked to use their own mobile devices to access it and interact with it for at least 15 min. The tool was built with the GPT Builder using a single ChatGPT Plus subscription held by the organizing team and deployed as a Custom GPT hosted on the ChatGPT platform. Interacting with it did not require a paid subscription: participants accessed it through the shared public link from their own ChatGPT accounts, with no individual licensing or local infrastructure required. It was allowed to use the same device in pairs or small groups to interact with the chatbot. However, each participant was required to complete the CUQ-IT evaluation form after the interaction. Additional instructions were also provided for security and privacy reasons, with particular emphasis on not sharing personal information or medical data of real patients with the chatbot, as Custom GPTs are not designed to ensure compliance with GDPR requirements and are not classified or validated as medical devices in Europe.

### 2.5 Development of VaxSense: a custom GPT for vaccination counseling

A custom generative AI tool named VaxSense was developed using OpenAI's GPT Builder platform to serve as a vaccination counseling assistant for Italian residents (the tool is accessible at https://chatgpt.com/g/g-VVDwWMNd1-vaxsense). The chatbot was designed to support informed decision-making and address vaccine hesitancy through evidence-based empathetic communication.

VaxSense was configured with four core interaction principles: (i) empathetic tone acknowledging user concerns; (ii) evidence-based reasoning grounded in scientific literature; (iii) reassuring communication to address fears and misinformation; and (iv) cultural awareness accommodating diverse health beliefs in the Italian context. These principles, together with the tool's intended scope and communication style, were encoded in the Custom GPT's configuration instructions (system prompt) and reinforced by the curated knowledge base described below; no custom moderation layer was added beyond this configuration and the platform's standard safeguards. The full configuration instructions are available in the Supplementary materials.

The chatbot's functional capabilities included: explaining vaccination schedules across age groups and special populations (e.g., pregnant women, immunocompromised individuals); addressing vaccine hesitancy with factual safety data; providing personalized recommendations based on medical history and travel plans; debunking common myths referencing peer-reviewed evidence; and counseling on post-vaccination reactions.

The knowledge base was constructed from multiple authoritative sources. The Italian National Plan of Prevention through Vaccination 2023–2025 (PNPV 2023–2025) served as the primary reference for age-specific immunization schedules, ensuring recommendations reflected the available vaccinations for all target populations, from newborns to older adults (18). Clinical guidance on vaccine contraindications, precautions, and best practices was derived from the CDC's Advisory Committee on Immunization Practices (ACIP) guidelines (19). Given that a substantial portion of post-pandemic vaccine hesitancy is linked to misinformation about mRNA vaccines and their purported adverse effects, a comprehensive umbrella review of COVID-19 vaccine safety, feasibility, and effectiveness across diverse populations and medical conditions was incorporated to equip the tool with robust evidence to address such concerns (20). Finally, a meta-analysis of vaccine uptake strategies informed the chatbot's communication approach, integrating evidence on effective messaging, policy-level interventions, and behavioral strategies to promote vaccination acceptance (21). This multi-source architecture ensured alignment with current Italian public health recommendations and international best practices, while maintaining conversational accessibility for lay users.

### 2.6 Evaluation of a generative AI tool and psychometric validation of the Italian version of the CUQ (CUQ-IT)

After the workshop, the CUQ-IT developed during “phase i” of the study was used to evaluate the usability of the Custom GPT (VaxSense) designed and tested during the workshop. It was administered to participants with the same modalities of the questionnaire described in Section 2.3. The evaluations of this pilot test of the CUQ-IT were used to assess the validity and the reliability of the translated questionnaire within the target population.

### 2.7 Statistical analysis

Each participant's response form was archived, and the data were transcribed into an electronic database for subsequent statistical analyses. Responses were stratified by gender, age, and profession, while preserving participant anonymity. To investigate differences in AI literacy, we used the Wilcoxon and Kruskal-Wallis tests. These non-parametric tests were selected because the AI-literacy items and CUQ-IT scores are ordinal and were compared between independent participant subgroups defined by gender, age, and profession, with small and unequal group sizes and without distributional assumptions. Given the pilot sample size and the number of subgroup comparisons performed, these analyses should be regarded as hypothesis-generating, and the corresponding *p*-values were not adjusted for multiple comparisons. The psychometric adequacy of CUQ was examined using the Kaiser-Meyer-Olkin (KMO) test and Bartlett's test of sphericity, followed by an exploratory factor analysis (EFA) using principal axis factoring with oblimin rotation; the number of factors was determined by parallel analysis, supported by the scree plot and the Kaiser criterion. CUQ's consistency was then assessed with Cronbach's alpha. Associations between KAP ratings and CUQ-IT scores were examined using Spearman's rank correlation. A *p*-value of 0.05 or less was considered indicative of statistical significance. As an exploratory pilot validation based on convenience sampling at a single workshop, no *a priori* sample size calculation was performed; sampling adequacy was instead evaluated *post hoc* through the KMO measure and the subject-to-item ratio. All statistical analyses were carried out using R software version 4.2.2.

## 3 Results

The workshop was attended by 150 participants, and 87 (58%) returned questionnaires. Of these, 1 was excluded due to missing demographic data. Of the 86 respondents eligible for the KAP survey on AI literacy, 9 (10.47%) did not complete the CUQ-IT, leaving 77 valid questionnaires for the usability chatbot analysis (89.53%). As reported in Table 1, participants were predominantly female (48/86, 55.82%). The mean age was 35.9 years old (SD = 8.7), with the most represented age group being 30–40 years old (53/86, 61.62%). Participants were mainly medical residents and PhD students (37/86, 43.02%).

**Table 1:** Results of the KAP survey on artificial intelligence among public health professionals, stratified by gender, age, and profession.

Participants	*n* (%)	AI knowledge		AI attitudes		AI practice	
		Mean ±SD	Median (IQR)	Mean ±SD	Median (IQR)	Mean ±SD	Median (IQR)
Overall	86 (100%)	2.74 ± 1.05	3 (2, 3)	3.87 ± 0.73	4 (3, 4)	2.88 ± 1.11	3 (2–4)
Gender		*p* = 0.006		*p* = 0.67		*p* = 0.68	
Men	37 (43.02%)	3.08 ± 1.01	3 (2–4)	3.89 ± 0.84	4 (3–5)	2.95 ± 1.10	3 (2–4)
Women	48 (55.82%)	2.46 ± 1.01	2 (2, 3)	3.85 ± 0.65	4 (3.75–4)	2.83 ± 1.14	3 (2–4)
Other	1 (1.16%)	4	4	4	4	3	3
Age group		*p* = 0.96		*p* = 0.22		*p* = 0.96	
18–30	13 (15.11%)	2.69 ± 1.11	2 (2, 3)	3.62 ± 0.51	4 (3, 4)	2.77 ± 1.09	3 (2–4)
30–40	53 (61.62%)	2.77 ± 1.15	3 (2, 3)	3.85 ± 0.84	4 (3, 4)	2.94 ± 1.17	3 (2–4)
40–60	18 (20.93%)	2.72 ± 0.75	3 (2, 3)	4.11 ± 0.47	4 (4)	2.78 ± 1.00	3 (2–3.75)
>60	2 (2.32%)	2.5 ± 0.71	2.5 (2.25–2.75)	4 ± 0	4 (4)	3 ± 1.41	3 (2.5–3.5)
Profession		*p* = 0.13		*p* = 0.021		*p* = 0.009	
Medical residents and PhD students	37 (43.02%)	2.89 ± 0.99	3 (2, 3)	3.84 ± 0.60	4 (3, 4)	3.19 ± 1.05	3 (3, 4)
Medical specialists	36 (41.86%)	2.72 ± 1.06	3 (2, 3)	4.03 ± 0.88	4 (4, 5)	2.86 ± 1.07	3 (2–4)
Healthcare professionals	12 (13.95%)	2.17 ± 0.94	2 (1.75–3)	3.5 ± 0.52	3.5 (3, 4)	2 ± 1.04	2 (1–3)
Other	1 (1.16%)	5	5	4	4	3	3

### 3.1 Knowledge, attitudes and practice toward AI in public health

Table 1 presents participants' stratification according to demographic categories and their responses to the KAP survey.

*Knowledge—How familiar are you with the application of AI in the healthcare domain?*

In the overall sample (n. 86), the mean AI knowledge score in healthcare was 2.74 (± 1.05), with a median of 3 (IQR = 1). Male participants reported higher self-rated familiarity than females (3.08 ± 1.01 vs. 2.46 ± 1.01; Table 1), and the Wilcoxon test indicated a statistically significant gender difference (*p* = 0.006), although this finding should be interpreted with caution given the single-item measurement and absence of correction for multiple comparisons. Across age groups, values ranged from 2.69 ± 1.11 in the 18–30 group to 2.77 ± 1.15 in the 30–40 group, with marginally lower values in older participants; however, the Kruskal-Wallis test did not reveal statistically significant differences across age strata (*p*-value **>** 0.05). By profession, medical residents and PhD students reported the highest knowledge (2.89 ± 0.99), while healthcare professionals reported the lowest (2.45 ± 0.99); again, no statistically significant differences emerged across professional categories (Kruskal-Wallis, *p*-value **>** 0.05; Table 1).

*Attitudes—How would you evaluate the use of AI in healthcare?*

Regarding attitudes toward AI in healthcare (Table 1), the overall mean was 3.87 (±0.73), with a median of 4 (IQR = 0.75). Male and female participants reported similar values (3.89 ± 0.84 vs. 3.85 ± 0.65; both medians = 4). Among the age groups, the most positive attitudes were observed in the 40–60 age group (4.11 ± 0.47) and among those over 60 (mean = 4). By profession, medical specialists expressed the most positive attitudes (4.03 ± 0.88), followed by medical residents and PhD students (3.84 ± 0.60).

*Practice—How frequently do you use AI-based tools in routine professional healthcare activities?*

Regarding AI-related practice (Table 1), the overall mean score was 2.88 (±1.11), with a median of 3 (IQR = 2). Male participants reported higher average AI use than female participants. Among the age groups, the over-60s reported the highest mean of 3 (± 1.41), followed by the 30–40 age group, with a mean of 2.94 (± 1.17). By profession, medical residents and PhD students reported the highest values, with a mean of 3.19 (±1.05), whereas other healthcare professionals reported the lowest value, with a mean of 2 (±1.04).

### 3.2 Validation of the Italian version of the CUQ

In the evaluation of the Italian adaptation of the CUQ, the factorability analysis indicated good sampling adequacy (KMO = 0.81) and significant correlations among items (Bartlett's test of sphericity: chi-square (120) = 515.36, *p* < 0.001). Exploratory factor analysis (principal axis factoring with oblimin rotation) was used to examine dimensionality. Parallel analysis and inspection of the scree plot indicated a single dominant factor: the first eigenvalue (6.09) accounted for 38.1% of the variance and was the only one exceeding its parallel-analysis threshold, while subsequent eigenvalues (1.48, 1.34, 1.07) fell at or below random expectation. In the single-factor solution all items loaded positively on a general usability dimension (loadings 0.31–0.76; 13 of 16 items at or above 0.40; the extracted factor explained 34.4% of the variance; Table 2), supporting the use of the CUQ-IT as a unidimensional total usability score, consistent with the instrument's original scoring as a single 0 to 100 index. Overall internal consistency was high (Cronbach's alpha = 0.881). For comparability with the conceptual domains of the original CUQ, items were additionally grouped into Personality, Responses handling, and Navigation ease, with subscale reliabilities of alpha = 0.697, 0.828, and 0.716, respectively. Given the subject-to-item ratio (approximately 4.8:1), the factor structure should be considered preliminary and confirmed through confirmatory factor analysis in larger samples. Table 2 presents the CUQ-IT items, their corresponding CUQ items, conceptual domains, and single-factor loadings with communalities (the complete CUQ-IT is available in the Supplementary materials).

**Table 2:** CUQ-IT items, corresponding original CUQ items, conceptual domains, and single-factor results.

#	Item	Domain	Load	*h^2^*
1	The chatbot's personality was realistic and engaging (ENG) La personalità del chatbot era realistica e coinvolgente (ITA)	Personality	0.57	0.32
2	The chatbot seemed too robotic (ENG) Il chatbot sembrava troppo robotico (ITA)	Personality	0.48	0.23
3	The chatbot was welcoming during initial setup (ENG) Il chatbot è stato accogliente durante il setup (ITA)	Personality	0.41	0.17
4	The chatbot seemed very unfriendly (ENG) Il chatbot è apparso molto freddo e distante (ITA)	Personality	0.31	0.10
5	The chatbot explained its scope and purpose well (ENG) Il chatbot ha spiegato bene il suo scopo e la sua funzione (ITA)	Personality	0.57	0.32
6	The chatbot gave no indication as to its purpose (ENG) Il chatbot non ha dato alcuna indicazione sul suo scopo (ITA)	Personality	0.38	0.14
7	The chatbot was easy to navigate (ENG) Il chatbot era semplice da usare (ITA)	Navigation ease	0.76	0.58
8	It would be easy to get confused when using the chatbot (ENG) Era facile confondersi usando il chatbot (ITA)	Navigation ease	0.36	0.13
9	The chatbot understood me well (ENG) Il chatbot ha compreso bene ciò che chiedevo (ITA)	Responses handling	0.70	0.50
10	The chatbot failed to recognize a lot of my inputs (ENG) Il chatbot non ha riconosciuto molti dei miei input (ITA)	Responses handling	0.57	0.33
11	Chatbot responses were useful, appropriate, and informative (ENG) Le risposte del chatbot erano utili, pertinenti e informative (ITA)	Responses handling	0.72	0.52
12	Chatbot responses were irrelevant (ENG) Le risposte del chatbot erano fuori contesto (ITA)	Responses handling	0.71	0.51
13	The chatbot coped well with any errors or mistakes (ENG) Il chatbot ha gestito bene eventuali errori o imprevisti (ITA)	Responses handling	0.55	0.31
14	The chatbot seemed unable to handle any errors (ENG) Il chatbot non sembrava capace di affrontare gli errori (ITA)	Responses handling	0.71	0.50
15	The chatbot was very easy to use (ENG) Il chatbot era davvero intuitivo (ITA)	Navigation ease	0.64	0.41
16	The chatbot was very complex (ENG) Il chatbot risultava troppo complicato (ITA)	Navigation ease	0.67	0.45

Load = factor loading; *h*^2^ = communality; principal axis factoring (negatively worded items reverse-scored).

### 3.3 Evaluation of the usability of the custom GPT developed during the workshop with the Italian CUQ

The CUQ-IT scores assigned to the Custom GPT VaxSense by participants (n. 77) ranged from 18.75 to 95.31 (Table 3). The mean score was 66.60 (SD = 15.29), with a median of 68.75 and an IQR of 15.62 (59.38–75). Male participants reported higher CUQ-IT scores (mean = 72.12, SD = 13.91, *p* = 0.004). By age, the highest positive evaluations were observed among the 30–40 year-olds (mean = 68.26, SD = 13.41); the youngest group gave the lowest ratings (mean= 61.98, SD = 19.90). Across professions, medical specialists (mean = 67.80, SD = 16.95) and medical residents and PhD students (mean = 66.75, SD = 15.33) reported the highest scores. Participants reporting advanced knowledge of AI (Table 4) had the highest CUQ-IT scores (mean = 74.11 ±14.17; median = 73.44). Regarding attitudes toward AI, participants who rated its use in healthcare very positively reported the highest scores (mean = 75.39 ± 14.49; median = 75.00), followed by those with positive evaluations. Participants who expressed negative or very negative attitudes toward AI reported the lowest CUQ-IT scores. Similarly, users who reported the highest AI use assigned the highest CUQ-IT score to VaxSense (76.82 ± 10.43). Across the full sample, only attitudes were significantly correlated with usability scores (rho = 0.27, *p* = 0.018); correlations with self-rated familiarity (rho = 0.15, *p* = 0.19) and frequency of use (rho = 0.19, *p* = 0.09) were not significant.

**Table 3:** Evaluation of a vaccination counseling chatbot (VaxSense) by public health professionals using the CUQ-IT stratified by gender, age, and profession.

Participants	VaxSense CUQ-IT score		
	Range	Mean ±SD	Median (IQR)
Overall	18.75–95.31	66.60 ± 15.29	68.75 (57.81–75.00)
Gender	*p* = 0.004		
Men	39.06–95.31	72.12 ± 13.91	73.44 (64.06–81.64)
Women	18.75–93.75	62.54 ± 15.29	64.84 (55.86–70.7)
Other	68.75	68.75	68.75
Age group	*p* = 0.71		
18–30	18.75–92.18	61.98 ± 19.9	65.63 (48.43–73.43)
30–40	39.06–95.31	68.26 ± 13.41	68 (59–75)
40–60	18.75–90.62	65.16 ± 16.83	70.31 (60.94–75)
Profession	*p* = 0.46		
Medical residents and PhD students	18.75–95.31	66.74 ± 15.34	68.75 (58.59–75.00)
Medical specialists	18.75–95.31	67.80 ± 16.95	70.31 (60.15–79.68)
Healthcare professionals	48.44–76.56	62.78 ± 9.70	65.63 (55.47–68.75)

**Table 4:** CUQ-IT scores of a vaccination counseling chatbot (VaxSense), stratified by participants' AI knowledge, attitudes, and practice.

Participants	VaxSense CUQ-IT score	
	Mean ±SD	Median (IQR)
Overall	66.60 ± 15.29	68.75 (57.81–75.00)
Knowledge	ρ = 0.15, *p* = 0.19
No knowledge (1)	66.52 ± 11.51	67.19 (58.59–74.22)
Vague knowledge (2)	63.88 ± 15.32	64.84 (55.86–73.44)
Basic knowledge (3)	67.40 ± 16.62	70.31 (61.33–75.00)
Good knowledge (4)	65.85 ± 14.53	68.75 (60.16–75.78)
Direct or advanced experience (5)	74.11 ± 14.17	73.44 (64.06–82.81)
Attitudes	ρ = 0.27, *p* = 0.018
Very negative (1)	39.06	39.06 (39.06–39.06)
Negative (2)	56.25	56.25 (56.25–56.25)
Neutral (3)	64.50 ± 12.76	66.41 (50.78–73.44)
Positive (4)	65.94 ± 15.66	68.75 (60.94–73.44)
Very positive (5)	75.39 ± 14.49	75.00 (69.14–87.89)
Practice	ρ = 0.19, *p* = 0.09
Never (1)	62.97 ± 15.40	65.62 (52.34–68.36)
Rarely (2)	63.72 ± 11.09	64.06 (53.91–73.05)
Occasionally (3)	68.69 ± 16.35	70.31 (62.89–80.47)
Often (4)	64.98 ± 18.10	64.06 (57.81–73.44)
Very often (5)	76.82 ± 10.43	74.22 (71.09-79.69)

## 4 Discussion

This study aimed to assess AI literacy among a sample of Italian public health professionals participating in a targeted educational workshop and to evaluate the usability of a custom-built Large Language Model (LLM) chatbot using the newly translated Italian version of the Chatbot Usability Questionnaire (CUQ-IT). Our findings offer two preliminary contributions: they provide an initial snapshot of AI readiness among Italian public health professionals and present a first validated Italian adaptation of a dedicated chatbot usability instrument (4, 5, 22).

Participants reported moderate knowledge of AI, positive attitudes, but limited practical use. A notable pattern emerging from our survey is the discrepancy between participants' positive attitudes toward AI and their limited practical application of these tools in their daily routine. While the mean attitude score was high, indicating a widespread recognition of AI's potential to transform public health, the practice score remained low. This pattern mirrors international trends observed in similar cohorts (23). Some authors reported that while trainee doctors in the UK acknowledged the transformative value of AI, they expressed reluctance to integrate it into clinical workflows due to a lack of formal training and institutional guidelines (23). This “attitude-practice gap” suggests that the barrier to adoption is not skepticism, but rather a lack of operational know-how (24). Professionals are ready to embrace the AI conceptually, but they lack the technical agency to do so. This underscores the urgency of moving beyond theoretical lectures toward hands-on training models, such as the workshop described in this study, which enable the transition from “willing to use” to “able to use.”

The workshop bridged theoretical foundations with hands-on experience, an approach increasingly recognized as essential for fostering meaningful AI literacy (24). Practical engagement with conversational agents has been shown to increase confidence, enhance perceived usefulness, and reduce skepticism toward generative AI (25, 26). This aligns with our observation that participants with higher pre-existing familiarity with AI rated chatbot usability more positively. Such associations are consistent with technology acceptance models in digital health, where familiarity, perceived ease of use, and digital self-efficacy strongly influence user satisfaction and adoption intentions (27).

A notable finding concerns gender disparities in AI knowledge, with male participants reporting significantly higher self-rated familiarity. This gap persisted in CUQ-IT evaluations, where males assigned higher usability scores to VaxSense. Similar gender disparities have been documented in digital competence and AI self-efficacy across clinical and educational settings (28, 29). Importantly, prior work suggests that such disparities often reflect differential exposure rather than intrinsic ability, and that hands-on practice and guided training may help narrow the gap, especially among female learners (30). This underscores the need for equitable and inclusive AI training strategies in public health education.

The evaluation of the Custom GPT VaxSense chatbot revealed good usability, with mean CUQ-IT scores comparable to or higher than those reported in recent studies assessing health chatbots and LLM-based clinical tools (31, 32). Participants with stronger AI attitudes and higher practice scores tended to evaluate usability more favorably, reinforcing the role of familiarity in shaping perceptions of chatbot intelligence, reliability, and communicative quality. Given the growing interest in conversational AI for public health communication and clinical support, such as vaccination guidance, travel medicine advice, and patient education, ensuring that these tools are both usable and trustworthy is essential for safe and effective implementation (33–35).

Another contribution of this study is the validation of the CUQ-IT, the first Italian adaptation of a dedicated chatbot usability questionnaire. The instrument demonstrated strong internal consistency, comparable to that of the original English validation (14). Exploratory factor analysis supported a single dominant usability dimension, consistent with the instrument's original scoring as a single total index; for comparability with the original CUQ, the items were additionally grouped into three conceptual domains (Personality, Response Handling, and Navigation Ease). The slightly lower reliability observed in the Personality domain is noteworthy but expected in a public health context. Unlike customer service chatbots designed to be “chatty” or empathetic, a public health tool like VaxSense prioritizes neutrality and accuracy. Users may therefore find questions about the chatbot's “engaging personality” less relevant or more difficult to interpret. Nevertheless, the CUQ-IT is a robust tool that fills a gap in the availability of non-English instruments for evaluating conversational agents.

Taken together, the findings highlight the potential of structured training that combines theoretical education, prompt engineering exercises, and hands-on chatbot development to strengthen AI literacy and foster practical skills among public health professionals (36). Pre-post studies integrating generative AI into health professional curricula have demonstrated substantial knowledge gains, and educational interventions have significantly improved understanding and attitudes toward AI in clinical contexts (37, 38). This educational model aligns with recommendations from the WHO (4) and the OECD (5), as well as recent proposals to integrate AI competencies into health curricula (4, 5, 39). The observed associations among literacy, attitudes, and usability suggest that AI training not only improves knowledge but also enhances users' readiness to evaluate and interact with AI-powered tools, a crucial competency for the safe and effective implementation of generative AI in public health practice.

Several limitations should be acknowledged. First, the use of convenience sampling within an AI-focused workshop likely introduced a selection bias, as participants were presumably more interested in technology than the general medical population. Relatedly, the sample was skewed toward early-career professionals, with medical residents and PhD students representing the largest subgroup (43.02%). This overrepresentation may have influenced both the baseline AI knowledge and attitudes observed, as younger professionals in training tend to report greater familiarity with digital technologies and generative AI tools compared to the broader public health workforce (23, 24, 29). Consequently, the KAP estimates reported here should not be interpreted as representative of the entire Italian public health community, and future studies should employ stratified sampling to ensure adequate representation across career stages and professional roles. Second, the small sample size, while sufficient for initial psychometric validation, has limited the generalizability of subgroup analyses, particularly for the over-60 age group; the resulting subject-to-item ratio (approximately 4.8:1) is modest for factor analysis, and the KAP constructs were each measured by a single self-report item, both of which constrain the psychometric conclusions.

Lastly, the cross-sectional design captured a snapshot of literacy and usability but did not allow us to assess the long-term retention of the skills acquired during the workshop. Moreover, the absence of a pre-post quantitative comparison limited the ability to formally quantify learning gains attributable to the workshop. Finally, the study evaluated the usability of VaxSense rather than the clinical accuracy of its outputs: the factual accuracy, appropriateness, and consistency of the chatbot's responses were not formally assessed by domain experts, and such evaluation would be required before any real-world deployment (10, 32).

## 5 Conclusion

This study can be considered as a contribution to the advancement of responsible and effective AI integration in public health in Italy. To begin with, it provides a psychometrically validated instrument for assessing chatbot usability in Italy, offering a standardized tool to evaluate AI-driven conversational agents. Second, it offers a snapshot of AI literacy among Italian public health professionals, revealing a pattern of cautious optimism coupled with limited practical experience, and highlighting a significant gender gap in self-reported AI knowledge.

Our findings support the adoption of integrated, hands-on educational models that combine theoretical AI foundations, prompt engineering, and critical evaluation exercises as a cornerstone of public health workforce development.

Future efforts should focus on scaling up such training initiatives and embedding them in formal postgraduate and continuing education curricula. Moreover, targeted strategies are needed to ensure that these opportunities are equitable and to effectively address existing literacy disparities, particularly those related to gender.

Empowering the public health workforce to competently use, critique, and co-design AI tools is no longer optional; it is a fundamental prerequisite for harnessing the potential of digital transformation while safeguarding against its risks.

## Data Availability

The data that support the findings of this study are available from the corresponding author upon reasonable request.
