# Supplementary material for: Training the public health workforce for generative AI: validation of the Italian Chatbot Usability Questionnaire
Source: Front Public Health. 2026 Aug 10;14:1860473. doi: 10.3389/fpubh.2026.1860473 (PMC13500654; doi:10.3389/fpubh.2026.1860473)
Supplement: Supplementary file 1 [file Data_Sheet_1.pdf]

Questionario sull'Usabilità dei Chatbot (CUQ-IT)

Grazie per aver scaricato la versione italiana del *Chatbot Usability Questionnaire*, uno strumento appositamente sviluppato per misurare l'usabilità dei chatbot.

Si prega di leggere attentamente queste istruzioni prima di utilizzare il questionario.

Il CUQ è composto da 16 domande strutturate in modo bilanciato, relative a diversi aspetti dell'usabilità dei chatbot. Otto di queste indagano gli aspetti positivi dell'usabilità del chatbot e otto gli aspetti negativi. I punteggi sono calcolati su una scala da 0 a 100. Per informazioni su come calcolare i punteggi del CUQ, consultare la sezione "**Calcolo del punteggio**" di questa guida. Il questionario può essere somministrato in formato cartaceo; tuttavia, per comodità, è possibile digitalizzarlo qualora si desideri utilizzare una piattaforma web. Si consiglia di consultare la guida utente della piattaforma scelta per determinare il modo più idoneo per compilare il questionario.

Come utilizzare il CUQ

Tutte e sedici le domande vengono valutate tramite una scala Likert a 5 punti. Le domande con numero dispari riguardano aspetti positivi del chatbot, mentre quelle con numero pari riguardano aspetti negativi. I partecipanti dovrebbero leggere attentamente ciascuna affermazione e decidere in che misura sono d'accordo con essa. Successivamente, dovrebbero indicare il proprio livello di accordo apponendo un segno di spunta (✓) o una croce (✗) nel cerchio che meglio rappresenta come si sentono rispetto all'affermazione.

<u>Esempio</u>

	Fortemente in disaccordo<br>1	In disaccordo<br>2	Neutrale<br>3	D'accordo<br>4	Fortemente d'accordo<br>5
**La personalità del chatbot era realistica e coinvolgente**	◯	◯	◯	◯	⊗

Nell'esempio sopra, il rispondente era fortemente d'accordo con l'affermazione "*La personalità del chatbot era realistica e coinvolgente*"; pertanto ha contrassegnato il cerchio numero 5 (completamente d'accordo).

Questionario sull'Usabilità dei Chatbot (CUQ-IT)

Per favore, completa questo questionario leggendo attentamente ogni affermazione e mettendo un segno di spunta (✓) o una croce (✗) nel cerchio che meglio corrisponde a come ti senti rispetto all'affermazione. Ricorda che non ci sono risposte giuste o sbagliate!

	Fortemente in disaccordo 1	In disaccordo 2	Neutrale 3	D'accordo 4	Fortemente d'accordo 5
**La personalità del chatbot era realistica e coinvolgente**	◯	◯	◯	◯	◯
**Il chatbot sembrava troppo robotico**	◯	◯	◯	◯	◯
**Il chatbot è stato accogliente durante il setup**	◯	◯	◯	◯	◯
**Il chatbot è apparso molto freddo e distante**	◯	◯	◯	◯	◯
**Il chatbot ha spiegato bene il suo scopo e la sua funzione**	◯	◯	◯	◯	◯
**Il chatbot non ha dato alcuna indicazione sul suo scopo**	◯	◯	◯	◯	◯
**Il chatbot era semplice da usare**	◯	◯	◯	◯	◯
**Era facile confondersi usando il chatbot**	◯	◯	◯	◯	◯
**Il chatbot ha compreso bene ciò che chiedevo**	◯	◯	◯	◯	◯
**Il chatbot non ha riconosciuto molti dei miei input**	◯	◯	◯	◯	◯
**Le risposte del chatbot erano utili, pertinenti e informative**	◯	◯	◯	◯	◯
**Le risposte del chatbot erano fuori contesto**	◯	◯	◯	◯	◯
**Il chatbot ha gestito bene eventuali errori o imprevisti**	◯	◯	◯	◯	◯
**Il chatbot non sembrava capace di affrontare gli errori**	◯	◯	◯	◯	◯
**Il chatbot era davvero intuitivo**	◯	◯	◯	◯	◯
**Il chatbot risultava troppo complicato**	◯	◯	◯	◯	◯

Calcolo del punteggio

NOTA: Per il calcolo del punteggio del CUQ-IT è possibile utilizzare lo stesso strumento usato per il CUQ: il *CUQ Calculation Tool* (disponibile sul sito web). Tuttavia, se si desidera effettuare il calcolo manualmente, si prega di seguire le istruzioni riportate di seguito (un esempio svolto è disponibile a pagina 4).

1. Per ciascuna domanda, assegnare un punteggio da 1 a 5 in base al livello di accordo con l'affermazione (ad es. "Fortemente d'accordo" vale 5 punti, "Neutro" vale 3 punti, "Fortemente in disaccordo" vale 1 punto).

2. Calcolare la somma di tutte le domande **dispari** (positive).

3. Calcolare la somma di tutte le domande **pari** (negative).

4. Sottrarre 8 dal punteggio ottenuto al punto 2.

5. Sottrarre dal valore 40 il punteggio ottenuto al punto 3.

6. Sommare i punteggi ottenuti ai punti 4 e 5. A questo punto si dovrebbe ottenere un punteggio su 64.

7. Dividere il punteggio ottenuto al punto 6 per 64 e moltiplicare il risultato per 100. Questo fornirà un punteggio CUQ su 100.

Poiché è probabile che il test venga condotto con più partecipanti, potrebbe essere utile calcolare il punteggio medio del CUQ, la mediana e così via. Si può fare ciò utilizzando il *CUQ Calculation Tool* oppure manualmente tramite un foglio di calcolo o un software statistico.

Questionario sull'Usabilità dei Chatbot (CUQ-IT)

Esempio

1. Si supponga che le domande del CUQ-IT siano state valutate come segue:

**Domanda**	**1**	**2**	**3**	**4**	**5**	**6**	**7**	**8**	**9**	**10**	**11**	**12**	**13**	**14**	**15**	**16**
Punteggio	4	4	5	1	4	4	5	1	4	1	4	1	3	2	5	1

2. Calcolare la somma di tutte le domande con numero dispari:
   4 + 5 + 4 + 5 + 4 + 4 + 3 + 5 = **34**

3. Calcolare la somma di tutte le domande con numero pari:
   4 + 1 + 4 + 1 + 1 + 1 + 2 + 1 = **15**

4. Sottrarre 8 dal risultato del punto (2):
   34 – 8 = **26**

5. Sottrarre il risultato del punto (3) da 40:
   40 – 15 = **25**

6. Sommare i risultati dei punti (4) e (5):
   26 + 25 = **51** (su 64)

7. Convertire il risultato del punto (6) in un punteggio su 100:
   (51 / 64) * 100 = **79,7**
